# Machine Learning in Polymeric Technical Textiles: A Review

**DOI:** 10.3390/polym17091172

**Published:** 2025-04-25

**Authors:** Ivan Malashin, Dmitry Martysyuk, Vadim Tynchenko, Andrei Gantimurov, Vladimir Nelyub, Aleksei Borodulin, Andrey Galinovsky

**Affiliations:** 1 AI Technology Scientific and Education Center, Bauman Moscow State Technical University, 105005 Moscow, Russia; dmart9945@mail.ru (D.M.);; 2 Scientific Department, Far Eastern Federal University, 690922 Vladivostok, Russia

**Keywords:** polymeric technical textiles, machine learning, artificial intelligence, smart materials, sustainable manufacturing

## Abstract

The integration of machine learning (ML) has begun to reshape the development of advanced polymeric materials used in technical textiles. Polymeric materials, with their versatile properties, are central to the performance of technical textiles across industries such as healthcare, aerospace, automotive, and construction. By utilizing ML and AI, researchers are now able to design and optimize polymers for specific applications more efficiently, predict their behavior under extreme conditions, and develop smart, responsive textiles that enhance functionality. This review highlights the transformative potential of ML in polymer-based textiles, enabling advancements in waste sorting (with classification accuracy of up to 100% for pure fibers), material design (predicting stiffness properties within 10% error), defect prediction (enabling proactive interventions in fabric production), and smart wearable systems (achieving response times as low as 192 ms for physiological monitoring). The integration of AI technologies drives sustainable innovation and enhances the functionality of textile products. Through case studies and examples, this review provides guidance for future research in the development of polymer-based technical textiles using AI and ML technologies.

## 1. Introduction

Polymeric materials are fundamental to the advancement of technical textiles, owing to their diverse properties such as flexibility [1], strength [2], and the capacity for tailored functionalization [3] (e.g., flame resistance, moisture management, and biocompatibility). Technical textiles [4,5] are fabrics engineered to meet specific performance criteria. They are utilized in sectors such as medical [6], defense [7], aerospace [8], and environmental [4] applications. Their production involves specialized manufacturing processes that combine principles from material science and textile engineering. This integration ensures that the fabrics achieve the predefined functional properties required by industry standards.

In recent years, the incorporation of machine learning (ML) and artificial intelligence (AI) into polymer research [9,10] has led to more efficient material design, enhanced performance prediction, and improved sustainability outcomes. The integration of polymer chemistry with AI and ML methodologies addresses the growing complexity of designing materials that fulfill stringent performance requirements [11,12], while also facilitating quality control in textile production. This approach has emerged in response to the increasing demand for innovative, high-performance technical textiles and represents a significant advancement in the field.

In the field of polymer materials, ML has been successfully applied in various domains, including property prediction (e.g., tensile strength [13], thermal stability [14], glass transition temperature [15]) based on polymer composition and structure. For example, Le et al. [16] presented a Gaussian process regression (GPR)-based ML model for predicting the tensile strength of polymer/CNT nanocomposites using 11 input parameters, achieving high accuracy with RMSE values of 5.982 MPa (training) and 5.327 MPa (testing), and MAE values of 3.447 MPa (training) and 3.539 MPa (testing).

Another promising area of ML-based applications in polymer textiles is the modeling of dyeing behavior [17], color fastness [18], and surface modifications [19] of polymer-based fabrics. Ant colony optimization (ACO) was applied by Souissi et al. [20] to predict optimal dye recipes for achieving uniform color across cotton and bicomponent polyester filament blends. The algorithm minimizes the color deviation between reactive dyeing of cotton and disperse dyeing of polyester, ensuring that both components achieve the same shade with minimal differences The developed algorithm is highly effective in minimizing the $\Delta_{\mathrm{ECMC}}$(2:1) values, ensuring that the color deviations between the two components of the fabric (bicomponent polyester filaments dyed with disperse dyes and cotton fibers dyed with reactive dyes) are minimized to acceptable levels. The iterative process involves ants exploring different dye concentrations, updating pheromone trails based on color matching success, and converging toward the best solution through a series of updates and probabilistic choices.

An additional promising application of ML in polymer textiles is the optimization of spinning and weaving parameters to control pore size, texture, and breathability in functional textile materials. Xie et al. [21] developed a novel artificial neural network (ANN) model to predict key mechanical properties such as breaking strength, elongation at break, and coefficient of variation (CV) of breaking strength, based on melt-spinning parameters like spinning temperature, take-roll speed, and metering pump speed. The model achieves prediction performance with a root mean square error (RMSE) of 0.3499, mean absolute percentage error (MAPE) of 0.0906, and coefficient of determination ($R^{2}$) of 2.9516.

ML-based applications in polymer textiles also include the prediction of degradation kinetics and aging behavior of biodegradable polymer fibers (e.g., PLA, PHB) under various environmental conditions. Wang et al. [22] investigated the accelerated aging behavior of glass/epoxy composites under hygrothermal conditions and compared it with naturally aged samples to assess the impact on tensile strength. After 3 years of natural aging, tensile strength decreased by 35.60%, while a 1000-h accelerated aging led to a 37.57% reduction. ML models, especially the random forest regressor, demonstrated the best performance in predicting natural aging times, with an $R^{2}$ value of 0.92, outperforming other models such as SVR.

Finally, smart textile development [23,24]—the integration of conductive polymer composites and ML algorithms for health monitoring, sensing, and adaptive responses in wearable systems—is an emerging frontier in ML applications within polymer textiles. The development of digitally enhanced fabrics is hindered by the lack of sustainable alternatives to metallic conductors, with material testing and optimization being time-consuming and resource-intensive. To address these issues, an ML-assisted approach was employed by Iannacchero et al. [25] to design fully textile-based conductive e-textile prototypes using Tencel yarn coated with polypyrrole. Through 11 experiments, the global optimum for reaction conditions was established, minimizing electrical resistance to 0.067 kΩ (22.3 Ωcm^−1^) and optimizing conductivity and cost-effectiveness using Bayesian optimization and Pareto front analysis. The optimized yarns were woven into prototype fabrics, demonstrating their potential in flexible, conductive wearable systems and heaters.

These developments are driven by the growing availability of experimental and simulation data, as well as the ability of ML algorithms to extract hidden patterns from high-dimensional datasets. Nevertheless, the application of ML in polymer science faces several challenges, including limited data availability, the need for physics-informed models, and generalization to unseen conditions. In this context, the integration of ML with domain knowledge, such as physics-informed neural networks (PINNs), has emerged as a promising strategy to overcome these limitations and advance the field of polymer engineering.

**Figure 1:**
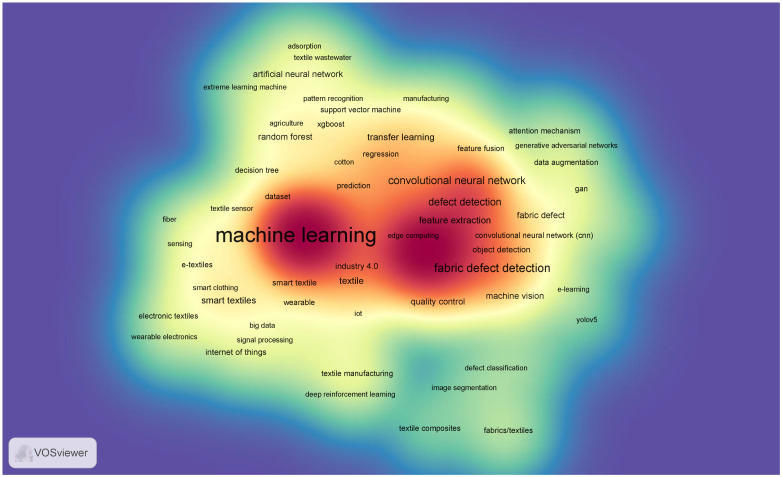
VOS density keyword map.

The VOS density analysis highlights (Figure 1) the growing role of ML and AI in polymer-based technical textiles, with a strong focus on optimizing material properties, manufacturing processes, and quality control. Key ML techniques such as artificial neural networks (ANN), convolutional neural networks (CNN), and ensemble learning are widely applied in polymer research for predictive modeling [26,27], defect detection [28,29], and process optimization [30]. The integration of AI in polymer textiles enhances smart textile development, enabling responsive and adaptive materials with applications in healthcare, wearables, and industrial sectors. The presence of terms like IoT, Industry 4.0, and feature fusion indicates a shift toward digitalized and automated textile production, improving efficiency and sustainability. AI-driven material design facilitates the discovery of novel polymeric compositions with enhanced durability, flexibility, and environmental resistance, supporting advancements in biodegradable and high-performance polymers. The dataset suggests an increasing focus on smart polymer textiles, defect detection, and AI-assisted material synthesis, paving the way for innovative, sustainable, and intelligent polymer-based textiles.

**Figure 2:**
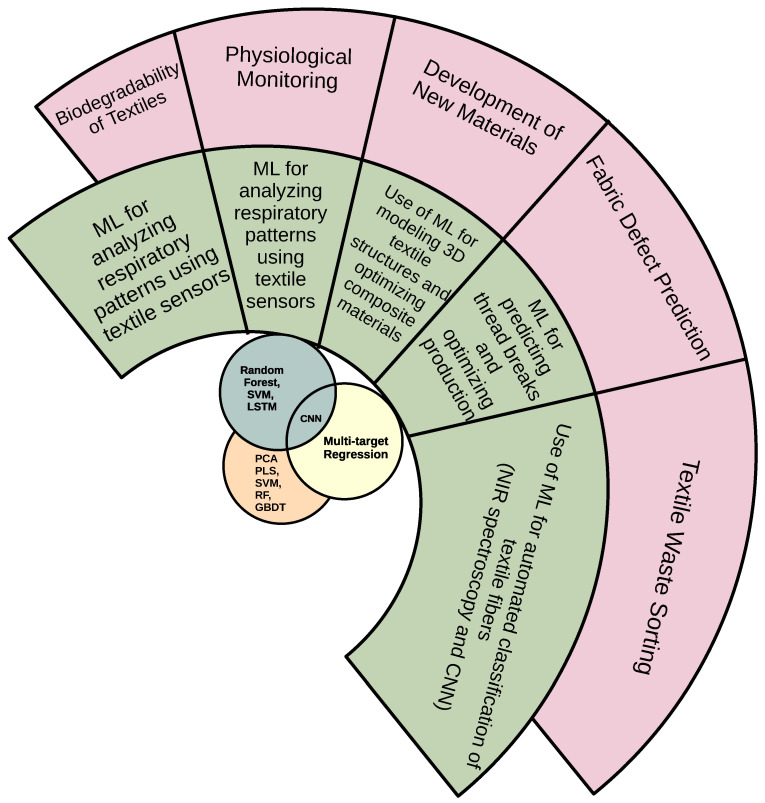
Key applications of ML in the textile industry (abbreviations: PCA—principal component analysis; PLS—partial least squares; SVM—support vector machine; RF—random forest; GBDT—gradient boosting decision tree).

Figure 2 is a circular diagram which highlights key ML applications in the textile industry. Textile waste sorting is essential for sustainability, utilizing ML for efficient fiber classification and reducing waste. Fabric defect prediction improves production efficiency by identifying potential issues like thread breaks, optimizing the manufacturing process. The development of new materials, including 3D textile structures [31]. Physiological monitoring through textile sensors enables the creation of smart fabrics that can track health parameters, offering significant potential in wearable tech. Lastly, modeling biodegradability in textiles plays a critical role in advancing environmentally friendly materials and reducing the ecological impact of the industry.

ML is increasingly applied to polymer-based technical textiles, offering advances in predictive design, process optimization, and sustainability. However, the existing literature often addresses AI in textiles or materials science in isolation, with limited focus on a distinct area. Cassola et al. [32] reviewed the current state of ML applications in polymer composites process simulation and explored relevant case studies in computational fluid dynamics, solid mechanics, and CAE to illustrate broader potential, summarizing key ML methods and their integration with engineering software tools. A review by Karuppusamy et al. [33] explored the growing role of ML in advancing polymer composite design and manufacturing, emphasizing its potential to improve material discovery, property prediction, and process optimization. Ge et al. [34] bridged polymer chemistry and ML, addressing challenges in dataset curation and model refinement while highlighting recent advances in polymer synthesis, modeling, and predictive applications. Their study emphasizes the need for interdisciplinary collaboration and discusses the importance of FAIR data principles and the integration of polymer theory with data-driven approaches.

This review examines the integration of ML in the development of polymeric materials for technical textiles, with a focus on on environmental aspects like textile recycling and the transition to a circular economy. The analysis addresses the contribution of these technologies to the optimization of polymer blends, enhancement of recyclability, and development of smart textiles with advanced functional capabilities. The incorporation of ML methods in polymer science is leading to measurable improvements in textile performance across various applications, including those in medicine, defense, and environmental protection.

## 2. Production Methods in Technical Textiles

In textile production, the fiber manufacturing process directly influences the structural and functional characteristics of the final material. Specialized techniques enable the creation of fibers with precise properties for specific applications. Two production processes—one for carbon fibers derived from polyacrylonitrile (PAN) [35] and another for fiberglass—demonstrate distinct approaches in textile manufacturing. These methods illustrate how different fiber production techniques shape the performance of textiles and determine their suitability for various industrial uses.

**Figure 3:**
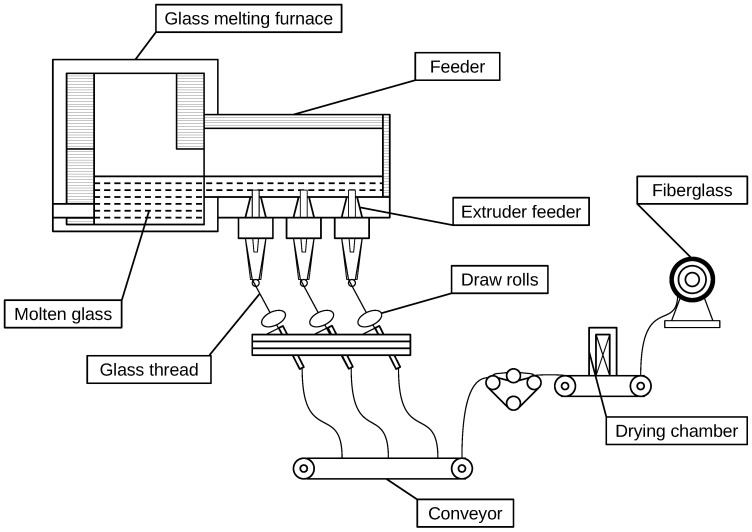
One-stage process for producing fiberglass, involving molten glass extrusion and filament solidification.

Figure 3 presents a one-stage process for producing fiberglass. In this method, glass is melted in a furnace until it forms a homogeneous molten state. The molten glass is then drawn into fine filaments through extruders. As the filaments exit the extruders, they encounter controlled temperature conditions that cause them to solidify. The solidified glass fibers are conveyed via a continuous conveyor system and wound onto a bobbin. This process results in glass fibers with uniform diameters suitable for applications in insulation, construction, and reinforcement.

**Figure 4:**
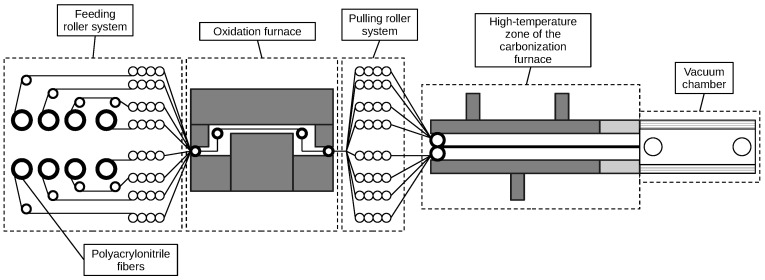
Combined oxidation and carbonization process of PAN fibers to produce high-quality carbon fibers.

Figure 4 illustrates the process of converting PAN fibers into carbon fibers through a combined oxidation and carbonization procedure. In this process, PAN fibers are introduced via feed rollers into an oxidation furnace. Here, the fibers are heated in the presence of oxygen to stabilize their molecular structure. Air circulation is maintained by a fan, and exhaust gases are removed through nozzles. The oxidized fibers are then transferred to a carbonization furnace, where they are subjected to high temperatures in an inert atmosphere created by the flow of inert gas. In some setups, a vacuum chamber is used to support the removal of non-carbon elements. The final step involves cooling the carbonized fibers and winding them onto receiving rollers for further processing.

Both processes illustrate the integration of thermal, chemical, and mechanical techniques to transform raw materials into functional textiles. The conversion of PAN fibers to carbon fibers and the extrusion-based production of fiberglass are essential for manufacturing textiles that meet specific performance requirements in various industrial applications.

Different fiber production processes significantly influence the final material’s performance by altering the fiber’s morphology, mechanical properties, and surface characteristics. For instance, melt spinning, with its high throughput and rapid cooling, typically produces fibers with superior tensile strength and uniformity, making it advantageous for large-scale, high-performance applications such as industrial textiles and reinforced composites [36]. In contrast, electrospinning yields nanofibers with high surface area, which is beneficial for applications requiring enhanced filtration [37], biomedical scaffolding [38], or functional coatings [39], although it generally operates at lower production rates. Wet spinning, by allowing controlled crystallization and fiber formation in a coagulation bath, can produce fibers with exceptional mechanical and optical properties, suited for high-quality specialty textiles [40]. Ultimately, the selection of a fiber production process depends on the specific industrial requirements: melt spinning is optimal for durability and mass production, electrospinning for applications demanding fine fiber morphology and high reactivity, and wet spinning for advanced composites where uniformity and multifunctionality are critical [41].

## 3. ML in Polymers-Based Textiles

In recent years, the textile industry has faced several environmental challenges related to high resource consumption and pollution [42,43]. Addressing these issues requires the implementation of sustainable technologies, including the shift toward a circular economy [44], process automation, and the use of new materials. One of the key areas is the application of ML and AI methods to improve textile waste sorting [45], predict fabric production defects, and develop innovative materials. This paper reviewed studies published between 2000 and 2025 on the use of these technologies in the textile industry, highlighting their role in improving production processes and reducing environmental impact.

The textile industry raises significant environmental concerns due to its linear production model, where textiles are produced, used, and discarded, contributing to resource depletion and pollution. A shift toward a circular economy requires efficient collection, classification, and recycling systems to recover high-quality fibers for reuse in new textile products. Riba et al. [46] proposed an automated classification method for post-consumer textile waste using near-infrared (NIR) spectroscopy and convolutional neural networks (CNNs) [47] to identify and separate textile fibers. A dataset of 370 samples, including pure fibers and binary mixtures, was analyzed, with CNN models achieving 100% accuracy for pure fibers and 90–100% for binary blends. The classification process involves preprocessing spectral data, applying dimensionality reduction methods, and training CNN models for pattern recognition. The results demonstrate that ML-based classification is effective for textile waste sorting and compatible with industrial-scale automation. This approach supports the transition to a sustainable textile industry by enabling precise fiber separation for high-value recycling.

Koptelov et al. [48] presented a deep learning (DL) approach to simulating 3D textile geometry, utilizing two different network architectures: convolutional and recurrent. These deep neural networks are trained to generate a fully compacted 3D textile unit cell based on the initial weave architecture, using precomputed weaving case studies. The method shows high computational efficiency, with predictions for stiffness properties accurate within 10% error. By leveraging AI, this approach enables rapid simulation of various textile architectures and their mechanical properties, helping to optimize composite material designs. Neural networks are trained using large datasets generated by kinematic weaving simulations to predict material behavior without needing detailed physics-based laws. The approach provides fast predictions, which is beneficial for real-time design and optimization tasks. Additionally, the study discusses the challenges of obtaining sufficient and diverse training data and explores solutions to ensure robust AI performance.

Respiration monitoring provides continuous assessment of physiological status and early detection of potential diseases. In the study by Huang et al. [49], a composite membrane consisting of polyacrylonitrile, carbon nanotubes, and latex (PCM) was developed to convert exhaled breath into a measurable current signal for respiratory rate and depth detection. The integration of ML enables real-time interpretation of sensor outputs, allowing the system to differentiate subtle variations in breathing patterns. ML algorithms process the sensor data to extract key features that correlate with respiratory anomalies, supporting early identification of conditions such as diabetes, wheezing, and obstructive sleep apnea syndrome. The latex encapsulation minimizes the impact of moisture and thermal fluctuations, ensuring stable and reliable sensor performance. Optimization of material composition, interdigital electrode design, and aperture area resulted in a response time of 192 ms and a recovery time of 104 ms. This approach demonstrates the potential of combining material design with ML to create a low-cost, efficient, and scalable wearable system for physiological monitoring and disease diagnosis.

Azevedo et al. [50] explored an ML approach for predicting faults in fabric production to minimize machine downtime. Their analysis focuses on a textile manufacturing environment operating within the Industry 4.0 framework, where client-specific customization impacts production planning and scheduling. Three regression tasks are addressed—predicting the number of weft breaks, warp breaks, and yarn bursts—enabling proactive interventions such as adjusting loom speed or modifying the sizing recipe. To automate model selection and optimization, several Automated ML tools, including $H_{2}$O, AutoGluon (version 1.0.0) [51], and AutoKeras [52], were utilized. A comparative analysis was conducted between single-target and multi-target regression strategies, with both direct and logarithm-transformed outputs. Experiments using IoT historical data from a textile manufacturer indicate that the single-target approach with H2O and logarithm-transformed data achieved the highest predictive performance, with an $R^{2}$ of 0.73 for weft breaks. Further evaluation using sensitivity analysis and explainable AI techniques demonstrated the potential of the approach to provide actionable insights for fault reduction in textile production.

Bao et al. [53] examined the degradability of polylactide (PLA) [54] and poly(hydroxybutyrate) (PHB) [55] blend fabrics in marine environments to assess their potential as sustainable bio-plastics.

The degradation of PLA/PHB-based fabrics primarily occurs through several key mechanisms: hydrolysis [56], microbial degradation [57], photo-oxidation [58], and thermal degradation [59]. Hydrolysis involves the cleavage of ester bonds in the polymer backbone through interaction with moisture, while microbial degradation relies on enzymatic activity of microorganisms capable of metabolizing PLA or PHB fragments. Photo-oxidative degradation occurs under exposure to ultraviolet (UV) radiation and oxygen, leading to chain scission, and thermal degradation results from elevated temperatures reducing the molecular weight of the polymers.

The rate and extent of PLA/PHB degradation are highly sensitive to external factors such as temperature, humidity, UV exposure, environmental medium, and polymer composition ratio. For example, increasing the ambient temperature from 25 °C to 58 °C can accelerate the hydrolysis rate of PLA by 6 to 8 times [60]. Similarly, at a relative humidity of 80%, the rate of hydrolysis can increase 2–3 times compared to dry conditions [61]. Prolonged exposure to UV radiation (over 300 h) may result in a 40% decrease in tensile strength due to photo-oxidative degradation [62].

Moreover, the type of environmental medium is a key factor in determining degradation efficiency. Under industrial composting conditions, PLA/PHB fabrics can reach up to 90% degradation within 60 days [53], whereas in soil environments, the degradation rate for the same period may not exceed 30%. The polymer ratio of PLA to PHB also significantly influences degradation behaviour. Increasing the PHB content by 20% has been shown to enhance biodegradation rates by 15–25%, while also affecting mechanical performance [63].

Key degradation mechanisms and quantitative effects of different conditions are summarized in Table 1.

Over an eight-week immersion period, PLA/PHB fabrics exhibited a gradual mass loss of 10.25–16.26%, with ultraviolet (UV) exposure and aeration accelerating abiotic hydrolysis. Structural changes in the polymers were analyzed using electrospray ionization mass spectrometry (ESI-MS), confirming degradation at the molecular level. In contrast, bulk PLA/PHB materials showed negligible mass loss, despite reductions in tensile strength, indicating the influence of fiber structure on degradability. To model and predict the nonlinear degradation behavior, an artificial neural network (ANN) was developed, utilizing a three-layer deep-learning architecture with rectified linear unit (ReLU) activation and backpropagation optimization. The ANN model effectively captured the impact of seawater conditions on mass loss, providing a predictive tool for evaluating biodegradation performance. The findings highlight the importance of material structure in marine degradation and suggest pathways for optimizing sustainable textile fibers.

Fiber-reinforced polymer (FRP) [64] composites are widely used for strengthening aging concrete structures, but their bond performance is significantly affected by high temperatures, especially during fire incidents. Salameh et al. [65] reviewed the degradation of bond strength between FRP systems and concrete substrates due to temperature exposure, analyzing both analytical and numerical bond–slip models. A key observation was that bond degradation accelerates when temperatures exceed the glass transition temperature of epoxy adhesives, while cement mortar-bonded carbon fiber-reinforced polymer (CFRP) [66] textiles demonstrate better fire resistance. To improve bond strength predictions, an ML model based on random forest regression was developed using experimental data from 37 beams. The model, trained on variables such as FRP elasticity, sheet dimensions, and test temperature, achieved an $R^{2}$ of 0.86, indicating high predictive accuracy. A parametric study confirmed the model’s ability to replicate real-world bond behavior under varying thermal conditions. This approach offers a data-driven alternative to extensive experimental testing, enhancing structural safety assessments in fire-prone environments.

Sinchuk et al. [67] investigated image segmentation methods for micro-computed tomography (μ-CT) data of CFRP composites with low contrast-to-noise ratios. The primary objective was to enable realistic geometry reconstruction for finite element modeling of textile composites at the meso-scale. Due to the minimal X-ray contrast between fibers and polymers and the unclear fiber boundaries, segmentation remains a challenge, particularly for low-resolution data where voxel size is comparable to fiber diameter. Two segmentation approaches were explored: a variational method and a DL-based U-Net neural network. The segmentation results were validated against a manually labeled dataset, serving as the ground truth. The DL approach achieved the highest segmentation accuracy, with a Dice similarity score of 4.7% voxel-wise deviation from the manual reference. These findings highlight the potential of DL for accurate and automated segmentation of CFRP composites, reducing reliance on manual processing and improving the efficiency of microstructural analysis.

Near-infrared (NIR) [68] spectroscopy is a tool for waste textile recycling, offering rapid, non-invasive, and both qualitative and quantitative analysis. However, moisture content in the textiles has been a major factor hindering the accuracy of NIR-based sorting. To address this, the external parameter orthogonalization (EPO) [69] algorithm was introduced by Qiu et al. [70] to minimize the interference from moisture, thereby improving the accuracy of NIR models. Various ML algorithms, such as PLS, ANN, RF, GBDT, SVM, and DL models like 1D-CNN were tested for their effectiveness with EPO preprocessing. The results showed that the EPO algorithm enhanced the NIR model’s accuracy, particularly in the presence of significant moisture interference, with an average $R^{2}$ score increase of 0.83. The study used 216 textile samples, with datasets representing different moisture levels, to develop and test the models. This approach demonstrated the potential of EPO in achieving more reliable waste textile sorting via NIR spectroscopy.

In the study by Gope et al. [71], AI algorithms were applied to optimize processing parameters for melt spinning machines, specifically targeting polypropylene (PP) [72] as the material. The study utilized a dataset containing 440 items to train a DL model for multi-quality optimization, focusing on factors such as screw and gear pump temperatures, screw speed, and take-up speed. The goal was to identify abnormal settings that led to subpar product quality, including issues like fineness, breaking strength, and elongation at break. The random forest algorithm was found to be highly effective in identifying abnormal parameter settings, with accuracy rates of up to 100% for certain classifications. Additionally, neural networks and ML techniques were compared, with a focus on optimizing the model to avoid overfitting and improve accuracy. Ultimately, the research presented methods that engineers can use to improve product quality and reduce manufacturing costs through precise control of processing parameters.

Kateb et al. [73] explored the development of textile-based capacitive strain sensors for wearable applications, focusing on the fabrication of highly sensitive strain sensors using conductive textiles. A conductive textile is created through vapor-phase polymerization (VPP) of pyrrole, optimized with methanol co-vapor and imidazole additives to improve conductivity. Insulation is achieved by coating the textile with thermoplastic polyurethane (TPU) [74] and a polystyrene-b block copolymer/barium titanate composite. These insulated conductive cords are then used to create capacitive strain sensors embedded in a textile glove. The system wirelessly measures capacitance changes in response to strain and monitors hand gestures, achieving 100% classification accuracy for 12 distinct gestures using ML. The study emphasizes the role of imidazole and methanol in enhancing the polymerization process, ensuring uniform conductivity across the textile. Additionally, the capacitive sensors exhibit high sensitivity and better gesture classification accuracy compared to resistive sensors.

Segmenting micro-computed tomography (μCT) [75] images of textile composites is crucial for mesoscale modeling but remains challenging for fiber bundle segmentation in carbon fiber reinforced composites. Traditional segmentation methods based on local fiber orientation struggle when tows have similar orientations or large touching areas relative to tow thickness. To address this, two new tow-splitting methodologies were proposed by Sinchuk et al. [76]: one based on geometrical analysis and the other leveraging DL to predict optimal watershed segmentation inputs. The DL approach is trained on synthetic woven composite images, eliminating the need for costly manual annotations. The study utilizes μCT scans of a plain-weave CFRP laminate, segmented using variational and DL methods. Instance segmentation is performed on five semantic segmentations, with the proposed methods successfully splitting compacted tows despite challenges like low contrast and noise. The results demonstrate that both approaches introduce less than 0.3% segmentation error, as measured against ground-truth data. These findings highlight the effectiveness of integrating DL with conventional image analysis for accurate tow segmentation in textile composites.

Using digital material twins allows for detailed examination of woven composite fabric architectures, fiber tow morphology, and defects, enabling analysis of mechanical performance, including damage and failure. A challenge in this process is the segmentation of low-contrast digital images, such as μCT or MRI images, and reconstructing 3D braided structures. To address this, a ResL-U-Net CNN [77] was proposed by Song et al. [78], incorporating a leaky-ReLU activation function for efficiency and a residual structure to prevent network degradation. Defects are implanted into the finite element model using three-dimensional spatial mapping, ensuring both authenticity and high-quality meshing. The study examines damage evolution and fracture features of GFRP material through digital material twins and verifies simulations with an in situ CT tensile experiment. The results indicate that digital twins accurately simulate mechanical performance, particularly in predicting damage locations and failure patterns. Image segmentation is performed using a U-Net DCNN, but limitations in accuracy necessitate the development of ResL-U-Net, which integrates a residual structure for improved robustness.

Sarkar et al. [79] focused on developing predictive models for the absorption properties of textile substrates treated with polyurethane (PU) and acrylic binder. The models were created using the adaptive neuro-fuzzy inference system (ANFIS) and artificial neural network (ANN) methods. The PU and acrylic binder were applied to dyed polyester knitted fabric, and the absorption percentage was taken as the output parameter. Two input parameters, binder (mL) and PU (%), were used in both models. The ANFIS model showed a coefficient of determination ($R^{2}$) of 0.98, while the ANN model had an $R^{2}$ of 0.93, indicating a strong fit between predicted and experimental data. The mean absolute error percentage (MAEP) was 0.76% for ANFIS and 1.18% for ANN, both well below the acceptable limit of 5%. The root mean square error (RMSE) for ANFIS was 0.61%, while it was 1.28% for ANN. These results demonstrate that both models are effective in predicting water absorption in PU-treated fabrics. The study also highlights the potential for these models to assist in the scalable production of functional textiles by reducing trial and error in the process. Future work could involve using larger datasets and exploring other binders and fabrics to improve model performance.

Gulihonenahali et al. [80] investigated the effect of giant reed fiber (*Arundo donax* L) reinforcement on the physical, mechanical, and thermal properties of polyethylene terephthalate (PET) composites. Composites were fabricated using compression molding with varying fiber loadings of 5 wt.%, 10 wt.%, and 20 wt.%. The optimal fiber concentration was found to be 10 wt.% (PET2), which exhibited maximum tensile strength of 5.4 MPa, flexural strength of 26 MPa, tensile modulus of 8343 MPa, and flexural modulus of 6300 MPa. The fibers, with a density of 693.25 kg/m^3^ and lengths between 18–22 cm, were extracted mechanically and used in their native form. Microstructural analysis using SEM revealed fiber pullout, gaps, and fracture behavior. Physical tests showed that water absorption increased with fiber content, while micro Vickers hardness was evaluated based on ASTM standards. Thermal characterization indicated enhanced thermal conductivity and flame resistance properties, with thermogravimetric analysis conducted between 35–600 °C. To optimize fiber loading for desired properties, an artificial neural network (ANN) model was developed, trained using 70% of data, and validated through k-fold cross-validation with 15% testing data. The ANN model demonstrated accuracy, precision, and robustness in predicting optimal fiber content, significantly reducing time and effort in experimental trials.

Madhavi et al. [81] investigated the mechanical behavior of textile-reinforced concrete (TRC) using different binder types: cementitious, geopolymer, and epoxy. A total of 12 mortar cubes (70.6 mm) and six cylinders (75 × 150 mm) were cast to analyze binder properties, while 84 large concrete cylinders (150 × 300 mm) were strengthened with three textile types: AR glass (ARG), basalt, and hybrid textiles. The cementitious binder showed superior 28-day compressive strength of 36.79 N/mm^2^, which was 25.26% higher than the geopolymer binder (27.49 N/mm^2^). Similarly, the split tensile strength of the cementitious binder (1.6 N/mm^2^) exceeded the geopolymer binder by 10.6%. Cylinders were strengthened using 2, 4, and 6 layers of textiles, and the hybrid textile combined with the cementitious binder exhibited the highest strength performance. Sandblasting of cylinders to a depth of 2–3 mm improved bonding conditions. Artificial neural network (ANN) modeling, with a three-layer architecture (six inputs, 10 hidden neurons, and one output), predicted TRC mechanical properties with high accuracy ($R^{2}$ > 0.99). ANN analysis optimized parameters affecting TRC performance, while SEM-EDX analysis confirmed enhanced C-S-H gel formation in cementitious binders, leading to better strength. The study concludes that TRC using cementitious binder and hybrid textiles is a promising solution for high-performance, durable concrete strengthening.

Jang et al. [82] investigated the impact of sewing thread patterns on the resistance behavior of silver-paste-coated conductive yarns. Conductive yarns were analyzed using SEM, revealing non-uniform silver particle distribution with gaps on the surface. Experiments varied stitch lengths from 1 mm to 5 mm and stitch angles from 180° to 20°, maintaining a fixed total stitch length of 10 cm and maximum thread tension. The results showed that shorter stitch lengths increased the yarn’s width, while stitch angles influenced resistance due to changes in the current flow path. Resistance was measured across 10 samples for each condition, collecting 1000 data points per sample. It was found that increased tension reduced resistance by decreasing the distance between silver particles. Multiple linear regression (MLR) and artificial neural networks (ANN) were used to model and predict resistance based on stitch parameters. While MLR captured linear relationships, ANN provided better accuracy by learning complex, nonlinear interactions. The study concludes that understanding stitch patterns allows optimization of resistance properties for wearable sensors and power transmission in smart textiles.

Amor et al. [83] investigated the prediction of tensile strength of nano titanium dioxide (${\mathrm{TiO}}_{2}$)-coated cotton using an artificial neural network (ANN) model trained with Bayesian regularization. The coating of cotton fabric (115 g/m^2^) was performed using UV radiation under varying conditions of ${\mathrm{TiO}}_{2}$ dosage (2–10 g/L), temperature (20–70 °C), and UV irradiation time (15–150 min). A total of 15 experimental samples were prepared. The developed ANN model consisted of three input nodes, two hidden layers with 12 neurons each, and two output nodes (${\mathrm{TiO}}_{2}$ coating amount and tensile strength). The ANN model outperformed multiple linear regression (MLR) and polynomial regression analysis (PRA) in prediction accuracy, achieving a mean absolute percentage error (MAPE) of 1.82% for tensile strength, compared to 2.75% for MLR and 4.12% for PRA. Additionally, the ANN model showed the highest $R^{2}$ value of 0.993 for tensile strength prediction, indicating a strong correlation between predicted and actual results. The root mean square error (RMSE) for tensile strength prediction was the lowest in ANN (3.12 N) compared to MLR (5.74 N) and PRA (9.51 N). SEM analysis confirmed uniform deposition of ${\mathrm{TiO}}_{2}$ nanoparticles on cotton surface after UV treatment. Overall, the study demonstrates that the ANN model is a highly accurate and effective tool for predicting the tensile strength of ${\mathrm{TiO}}_{2}$-coated cotton fabrics under varying processing conditions.

Kim et al. [84] proposed a convolutional neural network (CNN) model to predict the sheet resistance of conductive fabrics using brightness information extracted from scanned images. Conductive fabrics were fabricated using an eco-friendly bamboo double-sided fabric (60% bamboo, 40% cotton) with a dip-coating process using 0.1 wt.% water-based single-walled carbon nanotube (SWCNT) ink. A total of 10 fabric samples were prepared, with each 10 cm × 10 cm fabric undergoing various dip-coating cycles to induce diverse resistance levels. The scanned images (1440 × 1440 pixels) were preprocessed into grayscale and segmented into 250 smaller images (288 × 288 pixels) for CNN training. The dataset included image data paired with resistance values directly measured using a surface resistance meter on corresponding fabric sections. Statistical analysis (ANOVA) confirmed a significant correlation between image brightness and sheet resistance (*p*-value = 8.04145 × 10^−18^). The CNN model, trained using MATLAB’s Neural Network Toolbox, employed two convolution layers (with 10 filters each of sizes 8 × 8 and 16 × 16), batch normalization, ReLU activation, pooling layers, and dropout (20%) to prevent overfitting. The model demonstrated excellent predictive performance, achieving an RMSE of 0.0558 and an $R^{2}$ value of 0.9557, validating its feasibility for non-contact resistance prediction of conductive fabrics.

In the study by Razbin et al. [85], a novel approach was developed to predict the tensile behavior of polyamide-6 (PA-6) multi-ply yarns with an open-packing structure, combining geometrical analysis and artificial neural networks (ANNs). The model accounts for the deformation of fibers in different layers and calculates the number of fibers in each layer, incorporating gaps between layers. The model’s performance was validated using numerical analysis, showing high precision with an average $R^{2}$ value of 0.97 and a mean absolute percentage error (MAPE) of 4.65%. The geometrical analysis assumed that the monofilaments’ cross-sections were circular, their behavior under tensile loading was similar across layers, and the number of fibers per layer remained constant until the central monofilament ruptured. The tensile force on the yarn was calculated by summing the horizontal components of forces across layers, considering the twist angle of each layer and using Hooke’s law for strain-related force behavior. To model the nonlinear relationship between applied strain, strain rate, and force, a feed-forward ANN was employed, trained with 1176 data pairs split into 90% training and 10% testing. This network consisted of two input nodes, eight hidden nodes, and one output node, mapping from strain and strain rate to the force on a single monofilament. The tensile tests on the PA-6 monofilaments and multi-ply yarns were conducted under various strain rates and twist levels, with results confirming the model’s high accuracy. This model provides a robust, generalized method for predicting the tensile behavior of PA-6 multi-ply yarns, overcoming the limitations of traditional experimental and numerical methods.

The development of digitally enhanced fabrics is progressing, but challenges remain due to the lack of sustainable alternatives to metallic conductors. Testing and optimizing new materials for these fabrics is time-consuming and resource-intensive. The integration of wearable textiles with artificial intelligence (AI) systems [86] represents a rapidly advancing field at the intersection of materials science, electronics, and biomedical engineering. The combination of flexible textile-based sensors with ML algorithms enables real-time acquisition, processing, and interpretation of diverse physiological and biomechanical signals directly from the human body [87,88]. A notable example is the development of flexible textile-based hydrogen ($H_{2}$) sensors by Zhu et al. [89], fabricated via inkjet-printing reduced graphene oxide (rGO) with Pd nanoparticles onto textile substrates. These sensors exhibit a six-fold enhancement in sensing response compared to conventional polyimide-based sensors, while maintaining high sensitivity at room temperature. When integrated with ML-based data analysis and triboelectric energy harvesting for IoT-driven calibration of environmental parameters such as temperature and humidity [90], these systems enable reliable and autonomous environmental monitoring in wearable formats.

In addition to environmental sensing, key physiological parameters monitored by textile-AI systems include electrocardiographic (ECG) and electromyographic (EMG) signals, respiratory rate, body temperature, sweat composition, and motion dynamics. For example, Adams et al. [91] presents an AI-driven smart textile system for continuous, non-invasive cardiac monitoring, integrating bioimpedance spectroscopy (BIS) with ECG, SCG, PCG, and PPG sensors to synchronously capture over 110 cardiac parameters, with ML algorithms achieving high diagnostic accuracy in early detection of cardiac emergencies. Developed in collaboration with Fraunhofer IZM and Charité Berlin, the system utilizes biocompatible silicone-based sensors and optimized semi-dry textile electrodes embedded in a wearable vest, validated on clinical and PhysioNet datasets.

Such AI-assisted smart textile systems enable advanced applications in health monitoring and personalized medicine. In particular, wearable biosensors embedded in textile structures facilitate non-invasive, long-term tracking of vital signs in naturalistic settings [92]. ML techniques allow for dynamic pattern recognition [93], anomaly detection [94], and predictive analytics [95], enabling early identification of pathophysiological changes, optimized therapeutic interventions, and customized feedback to users. This paradigm is increasingly applied in remote patient monitoring [96], rehabilitation programs [97], elderly care [98], sports performance optimization [99], and chronic disease management [100].

To summarize the key studies in textile technology and ML applications, Table 2 provides an overview of the research approaches, methodologies, and findings. The table highlights how various ML techniques are being applied to solve critical issues in textile manufacturing, waste recycling, and material development, showcasing advancements in areas like automated waste sorting, fabric defect prediction, and real-time respiratory monitoring.

## 4. Overview of Key Findings and Insights

### 4.1. Limitations

The integration of ML and artificial intelligence (AI) in the textile industry offers significant potential in addressing environmental challenges, but several limitations remain in current research. Firstly, while the implementation of ML algorithms in textile waste sorting and defect prediction has demonstrated promising results, many of these studies are based on small, non-representative datasets, limiting their scalability and real-world applicability. For example, Riba et al. [46] achieved high accuracy in waste sorting with a limited dataset of 370 samples, which may not capture the variability of real-world textile waste. Additionally, DL approaches, while useful in simulating complex textile geometries and predicting material behaviors, often require large, diverse datasets and significant computational resources, making them difficult to implement at an industrial scale without substantial investment in infrastructure [48].

Moreover, challenges related to the performance and reliability of smart textile-based wearable sensors persist. While innovations in ML-powered health monitoring systems, such as those for cardiac diagnostics, show high potential, their reliance on accurate data collection from diverse physiological signals can be hindered by external factors like sensor noise, variability in skin conditions, and environmental interference. Studies such as that by Adams et al. [91] have demonstrated high diagnostic accuracy in controlled settings; however, the robustness and reliability of these systems in real-world, everyday conditions remain uncertain. Furthermore, the integration of wearable technologies with energy-harvesting methods, such as triboelectric systems, often faces challenges in ensuring long-term sustainability and minimal power consumption, which are critical for practical, continuous use in health monitoring applications [90].

Another limitation is the lack of sustainable and scalable materials for use in AI-driven smart textiles. While research on biodegradable and eco-friendly fibers is advancing, such as the development of PLA/PHB-based fabrics for marine environments [53], achieving the desired balance between material performance, cost, and environmental impact remains a significant challenge. Furthermore, although ML approaches are increasingly used to optimize the production of smart textiles, such as in the design of conductive fabrics [25], the absence of universally applicable, sustainable alternatives to traditional metallic conductors limits the full potential of these technologies.

### 4.2. Challenges

**Figure 5:**
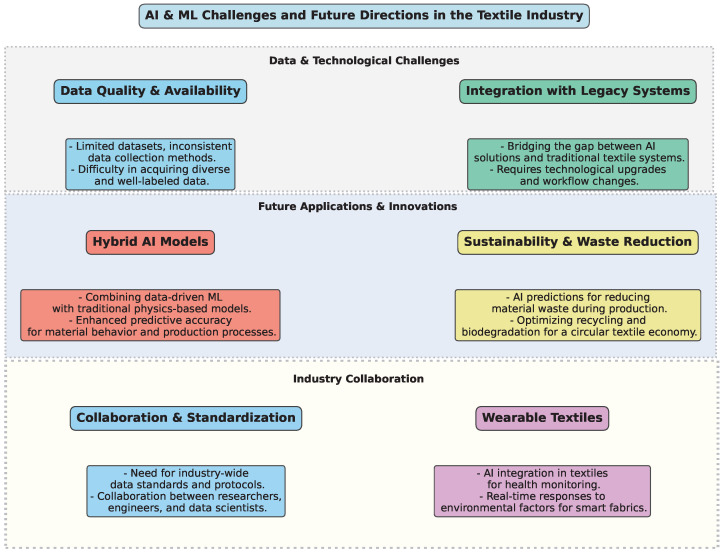
Key challenges and future directions for AI and ML in the textile industry.

The integration of AI and ML into the textile industry presents several challenges and opportunities that will shape the future of manufacturing, material development, and sustainability. Key obstacles include data quality issues, difficulties in integrating AI with legacy systems, and the need for standardized protocols. However, advancements in hybrid AI models, sustainability-focused AI applications, and wearable textiles offer promising directions. Ethical considerations and industry collaboration are also crucial to ensuring responsible AI adoption. Figure 5 illustrates the major challenges and future directions of AI and ML in the textile industry.

One major issue is data quality and availability; high-performance models depend on large, diverse, and well-labeled datasets, yet acquiring such data remains difficult due to the heterogeneous nature of textile materials and the variability in production processes [101]. In addition, the complex interplay of process parameters, material properties, and environmental factors creates a high-dimensional problem space that complicates model training and interpretation. This challenge is further exacerbated by the fact that many existing studies rely on controlled experimental data, which may not fully represent the variability encountered in industrial settings.

Another challenge lies in the seamless integration of AI solutions with traditional textile manufacturing systems. Many production environments are built around legacy equipment and processes that lack the digital infrastructure required for real-time data acquisition and processing [102]. Bridging this gap requires not only technological upgrades but also significant changes in workflow and operator training, making the transition to AI-driven systems both capital-intensive and disruptive.

### 4.3. Future Directions

Based on this review, ML has the potential to transform textile design and production by optimizing design parameters, automating quality control, and enhancing sustainability. For example, ML algorithms can predict material properties during the development phase, reducing the trial-and-error cycle and accelerating innovation [103]. In manufacturing, real-time data collection and process automation can be achieved through AI-driven monitoring systems, leading to improved production efficiency and reduced waste. These technologies can contribute to sustainable practices by optimizing resource use and minimizing environmental impact. However, challenges remain, including the need for large, high-quality datasets, the complexity of integrating multi-scale phenomena, and the adaptation of AI models to variable production environments. This interdisciplinary approach is expected to drive cost-effective and eco-friendly advancements in textile production.

The future of AI in the textile industry is incredibly promising, with areas of development that will transform the sector. A key focus is improving data collection and standardization, as ML models rely heavily on high-quality, standardized datasets. Currently, there is a lack of universal standards across the textile industry, with data often being inconsistent and difficult to compare [104]. With the advent of IoT and smart sensors, more data are being collected in real time, which can be used to improve ML models. However, to maximize the potential of these models, data must be standardized and of a high quality, which requires further work on creating universally accepted data benchmarks and improving data annotation processes.

Another exciting direction for the textile industry is the development of hybrid AI models that combine data-driven ML with traditional physics-based models [30]. These hybrid models can enhance the prediction accuracy of textile material behavior, as they use both empirical data and the principles of textile mechanics. This can be particularly valuable in the development of advanced materials and in quality control processes. Furthermore, hybrid models can optimize manufacturing processes, improving the overall efficiency of textile production and helping to reduce waste and energy consumption.

AI’s potential to drive sustainable practices within the textile industry is also a key area for future research. ML algorithms could be instrumental in reducing material waste during production by predicting the exact amount of raw material needed. AI can also be used to improve textile recycling processes, contributing to the shift toward a circular textile economy. The development of AI-driven models for biodegradation and material recovery would greatly enhance the environmental sustainability of textile manufacturing.

As the textile industry continues to evolve, we will also see greater automation and real-time process control driven by AI. Predictive maintenance, real-time quality control, and fault detection will become commonplace, significantly improving product consistency and reducing downtime. AI could also be used to optimize the handling, cutting, and stitching of textiles through industrial robots, boosting productivity and reducing human error. Real-time monitoring systems would help identify potential issues before they become major problems, reducing costs and improving efficiency.

The rise of wearable textiles and smart fabrics offers another exciting avenue for AI development. These fabrics, which are embedded with sensors, can monitor a variety of physiological and environmental conditions. AI algorithms can process data from these sensors, enabling real-time health monitoring and personalized care applications. Furthermore, AI could be used to optimize the functionality of wearable textiles, for instance, by adjusting the textile’s properties in response to environmental factors such as temperature or humidity. This integration of AI and wearable textiles could lead to significant innovations in fields like healthcare, sports, and fitness.

With the increased reliance on AI in decision-making processes within the textile industry [105], ethical considerations must be addressed. Data privacy, algorithmic fairness, and transparency are crucial factors that need to be prioritized. AI systems should be designed in ways that are explainable, transparent, and free from biases. As AI-driven decisions begin to affect worker safety, product quality, and environmental impact, ensuring the ethical deployment of AI is essential. Research focused on establishing ethical guidelines and transparent practices in AI development will be crucial for the responsible adoption of AI technologies in the textile sector.

Finally, rather than replacing human workers, AI in the textile industry will increasingly be used to augment human capabilities. AI systems will assist textile designers and engineers by providing real-time insights, optimizing designs, and improving production processes. This collaborative approach, where AI works alongside human expertise, will drive innovation and improve decision-making in the industry. The goal is not to replace workers but to enhance their ability to make informed decisions, boosting both creativity and efficiency.

In addition, there is a growing need for standardized protocols and benchmarks in textile-related AI applications to ensure that models developed in academic research can be reliably scaled and deployed in industrial environments. Collaboration across disciplines—bringing together experts in materials science, textile engineering, and data analytics—will be critical to developing integrated solutions that address both technical and operational challenges. As these advancements continue, they promise to drive not only improvements in manufacturing efficiency and product quality but also significant strides toward a more sustainable, circular textile economy.

## 5. Conclusions

ML is transforming polymer-based technical textiles by enabling predictive material design, optimizing manufacturing processes, and enhancing sustainability. However, several challenges and opportunities have emerged that need to be addressed for successful implementation. One significant hurdle is the quality and availability of data. High-performance ML models depend on large, diverse, and well-labeled datasets, which are often difficult to acquire in the textile industry due to the inherent heterogeneity of textile materials and the complexity of production processes [46,48].

Integrating AI with existing manufacturing infrastructure also presents obstacles, especially when legacy systems and outdated equipment are in place. Upgrading this infrastructure and training personnel involves high costs and risks disrupting established workflows [65,73]. In this context, improving data collection methods, standardizing data formats, and creating reliable benchmarks become essential steps to enhance AI model effectiveness—particularly as IoT and smart sensors are increasingly used to collect real-time data [70,71].

The development of hybrid models that blend ML with physics-based simulations is another promising avenue. These models can better predict textile material behavior, improve quality control, and optimize processes, all while minimizing waste and energy consumption [48,53]. Moreover, ML-driven innovations can optimize raw material usage, promote textile recycling, and aid in the development of biodegradable materials—contributing significantly to a circular textile economy [53,70].

AI’s role in automating and enhancing real-time control of production processes is also expected to expand. Predictive maintenance, fault detection, and optimization of tasks such as cutting, stitching, and handling will increase efficiency and reduce downtime [71,73]. Furthermore, AI is driving the integration of smart textiles and wearable technologies capable of monitoring physiological and environmental parameters. These innovations hold great promise for applications in healthcare, fitness, and sports, offering personalized and real-time care solutions [49,73].

To fully harness the potential of AI in textiles, interdisciplinary collaboration between material scientists, textile engineers, and data analysts is vital. Establishing standardized protocols and benchmarks will be key to scaling AI solutions across industrial settings [46,48]. Ultimately, the continued advancement of AI will not only enhance manufacturing efficiency and product quality but also steer the textile industry toward a more sustainable and circular future [70,73].

## Figures and Tables

**Table 1:** Influence of external factors on the degradation of PLA/PHB fabrics.

Factor	Influence on Degradation	Quantitative Data
Temperature	Accelerates hydrolysis and biodegradation	At 58 °C, the hydrolysis rate of PLA increases 6–8 times compared to 25 °C
Humidity	Activates hydrolysis	At 80% relative humidity, degradation proceeds 2–3 times faster
UV Radiation	Initiates photo-oxidation	After 300 h of UV exposure—tensile strength decreases by 40%
Medium type (compost, soil, aquatic environment)	Determines microbial activity	In compost, PLA/PHB degrades by 90% within 60 days; in soil—less than 30% over the same period
PLA/PHB ratio	Affects strength and degradation rate	Increasing PHB content by 20% accelerates biodegradation by 15–25%

**Table 2:** Summary of studies on textile technology and ML applications.

Study	Topic	Key Approach	Key Findings	Challenges	Potential Directions and Applications
Riba et al. (2022) [46]	Automated classification of textile waste	Near-infrared (NIR) spectroscopy & convolutional neural networks (CNNs)	100% accuracy for pure fibers, 90-100% for binary blends, supports recycling	Variability in textile blends and moisture content	Improve model robustness to diverse fabrics and industry adoption for waste sorting.
Koptelov et al. (2024) [48]	DL for simulating 3D textile geometry	Convolutional and recurrent neural networks	Efficient simulation of textile structures with 10% error in stiffness prediction	High computational cost for 3D simulations	Integration with real-world production environments for real-time simulations.
Huang et al. (2024) [49]	Breath monitoring using composite membrane	Composite membrane with polyacrylonitrile, carbon nanotubes, latex, and ML	Real-time respiratory monitoring with fast response and high sensitivity	Challenges in ensuring long-term stability and robustness of sensors	Expand sensor integration into wearables for health monitoring.
Azevedo et al. (2022) [50]	Predicting faults in fabric production	ML for predicting faults such as weft breaks, warp breaks, and yarn bursts	High predictive performance for faults, $R^{2}$ = 0.73 for weft breaks	Limited training data and variance in fault types	Explore advanced models for dynamic and real-time fault prediction.
Bao et al. (2022) [53]	Degradability of PLA/PHB blend fabrics in marine environments	Degradability testing and artificial neural network (ANN) modeling	ANN effectively predicts biodegradation behavior, material structure impacts degradation	Variability in degradation due to environmental factors	Extend research to other environmental conditions and enhance material designs.
Salameh et al. (2024) [65]	Bond performance of FRP composites in high temperatures	ML using random forest regression for bond strength prediction under thermal conditions	High predictive accuracy ($R^{2}$ = 0.86) for bond strength degradation in fire	Limited data on high-temperature behavior of materials	Apply findings to improve fire-resistant materials in construction.
Sinchuk et al. (2020) [67]	Image segmentation for CFRP composites	Variational and DL-based segmentation for low contrast data	DL achieved the highest segmentation accuracy for CFRP composites	Difficulty in segmenting low-contrast images due to noise	Enhance segmentation algorithms for use in automated manufacturing.
Qiu et al. (2023) [70]	NIR-based textile waste sorting with moisture interference	Orthogonalization of External Parameters (EPO) algorithm and various ML models	EPO improves accuracy of NIR sorting in moist textiles, $R^{2}$ score increase of 0.83	Moisture variation in textiles complicates sorting accuracy	Expand the method to broader types of textile waste sorting.
Gope et al. (2022) [71]	Optimizing melt spinning parameters for PP	DL and random forest models for identifying abnormal processing parameters	100% classification accuracy for abnormal settings, improved quality control	Challenge of integrating these models into real-time production systems	Future work could involve real-time processing monitoring in textile factories.
Kateb et al. (2024) [73]	Textile-based capacitive strain sensors	Capacitive sensors embedded in textiles using conductive textiles and ML for gesture recognition	100% accuracy in gesture classification, high sensitivity compared to resistive sensors	Integration of sensors with other wearable devices	Applications in healthcare and sports for real-time monitoring.
Sinchuk et al. (2021) [76]	Segmentation of textile composites in μCT images	Geometrical analysis and Deep Learning (DL) for tow-splitting in carbon fiber reinforced composites	Both methods reduce segmentation error to less than 0.3%, effectively splitting compacted tows despite challenges like low contrast and noise.	Noise and artifact removal from μCT images can still be challenging	Enhance the methods for wider industrial applications in composite materials.
Song et al. (2023) [78]	Digital material twins for woven composite fabric architectures	ResL-U-Net CNN with leaky-ReLU and residual structure to improve segmentation of low-contrast images	Digital twins accurately simulate mechanical performance, predicting damage locations and failure patterns with improved segmentation robustness.	High computational complexity in real-time simulations	Broader applications in composite material lifecycle monitoring and maintenance.
Iannacchero et al. (2025) [25]	ML for conductive textile prototypes	ML-assisted design with Bayesian optimization and Pareto front analysis for optimizing conductivity and cost	Optimal processing conditions were found for creating conductive fabrics; p-toluenesulfonic acid had minimal impact on conductivity.	Need for better materials to enhance conductivity and reduce costs	Applications in smart textile industries for sensors and energy harvesting.
Sarkar et al. (2021) [79]	Predictive modeling of textile absorption	Used ANFIS and ANN to predict water absorption in PU-treated polyester fabrics	ANFIS model had higher accuracy ($R^{2}$ = 0.98) compared to ANN ($R^{2}$ = 0.93), both effective in predicting water absorption.	Variability in textile treatments and fabric types	Expand modeling for other textile treatments and develop real-time monitoring systems for water absorption in textiles.
Gulihonenahali et al. (2022) [80]	PET composites with giant reed fiber	Used compression molding and ANN to optimize fiber loading (5%, 10%, 20%)	10% fiber content yielded optimal mechanical properties. ANN model accurately predicted fiber loading, reducing experimental trials.	Variability in fiber properties and molding conditions	Future work could focus on integrating fiber optimization into mass production and scaling the application of natural fibers in composites.
Madhavi et al. (2024) [81]	Mechanical behavior of Textile-Reinforced Concrete (TRC)	Compared cementitious, geopolymer, and epoxy binders with different textile reinforcements. ANN model used for property prediction.	Cementitious binder with hybrid textiles showed superior strength. ANN accurately predicted TRC properties with $R^{2}$ > 0.99.	Limited data on long-term behavior of TRC under different conditions	Extend research to include durability and environmental factors for optimized construction materials.
Jang et al. (2023) [82]	Resistance behavior of conductive yarns	Studied the impact of sewing thread patterns (stitch length, angle) on resistance using MLR and ANN.	Shorter stitch lengths reduced resistance. ANN provided better accuracy for predicting resistance.	Stitching complexity and variability in fabric constructions	Apply the findings to improve the performance of wearable electronics and smart textiles in real-world applications.
Amor et al. (2022) [83]	Prediction of tensile strength of ${\mathrm{TiO}}_{2}$-coated cotton	Used ANN to predict tensile strength of cotton coated with ${\mathrm{TiO}}_{2}$ under varying UV conditions.	ANN outperformed MLR and PRA with an $R^{2}$ of 0.993 and low error (MAPE = 1.82%) in predicting tensile strength.	Impact of UV degradation and surface coating uniformity	Explore extending this model to predict the performance of other coated textiles and in various environmental conditions.
Kim et al. (2024) [84]	Sheet resistance prediction in conductive fabrics	Developed a CNN model using brightness from scanned images to predict sheet resistance.	CNN model showed excellent performance with RMSE of 0.0558 and $R^{2}$ = 0.9557, accurately predicting resistance.	Variability in fabric characteristics and scanner resolution	Implement the model in real-time quality control systems in smart textile manufacturing.
Razbin et al. (2024) [85]	Tensile behavior of polyamide-6 yarns	Combined geometrical analysis and ANN to model tensile behavior of multi-ply yarns.	The ANN model achieved high accuracy ($R^{2}$ = 0.97, MAPE = 4.65%), providing an effective method for predicting tensile behavior.	Complexity of multi-ply yarn geometry and data acquisition	Develop advanced models to predict the behavior of different yarn constructions in various textile applications.
